# International Trade and Health in Thailand: A Scoping Review

**DOI:** 10.3390/ijerph182111692

**Published:** 2021-11-07

**Authors:** Kamonwan Kiewnin, Titaree Boontantrapiwat, Jeerapa Sosom, Mintar Hongtumrong, Anon Khunakorncharatphong, Churnrurtai Kanchanachitra, Cha-aim Pachanee

**Affiliations:** 1 International Health Policy Program, Ministry of Public Health, Nonthaburi 11000, Thailand; kamonwan@ihpp.thaigov.net (K.K.); jeerapa@ihpp.thaigov.net (J.S.); mintar@ihpp.thaigov.net (M.H.); anon@ihpp.thaigov.net (A.K.); 2 Faculty of Social Sciences and Humanities, Mahidol University, Nakhon Pathom 73170, Thailand; titaree.boo@mahidol.ac.th; 3 Institute for Population and Social Research, Mahidol University, Nakhon Pathom 73170, Thailand; churnrurtai.kan@mahidol.edu

**Keywords:** international trade, health, Thailand, policy, impact

## Abstract

International trade has become more complicated and is now related to more aspects of health and the health system. As Thailand is active in international trade and health, understanding what knowledge exists and determining the knowledge gap is essential for generating the necessary evidence in order to promote better understanding and allow evidence-based policy decisions to be made. This study reviewed the existence of knowledge on international trade and health issues in a scoping review, focusing on Thailand during the period 1991–2020. In total, 156 studies from seven databases and manual searching were included. Of these, 46% were related to trade in health services and 39% were linked to intellectual property, particularly access to medicines. This review found only a very small amount of research on other issues and did not identify any study on trade policies or products related to health and international trade and the environment. We therefore recommend that further studies should be carried out to provide more critical evidence—in particular, more research focusing on the impacts of trade on health-related goods and the analysis of the positive and negative impacts of international trade on industry is needed. Furthermore, better knowledge management through the publication of research findings and making them searchable on international databases will increase the visibility of international trade, increase our knowledge of health issues, and provide supporting evidence.

## 1. Introduction

International trade undeniably provides countries with enormous economic benefits in terms of the export and import of both goods and services, leading to an increase in national income. Consumers have more choices and thus are able to purchase their preferred products or services at a lower price [1]. Despite its benefits, international trade can negatively affect the health of populations as well as exacerbate the problems of poverty, inequality, and environmental damage [2]. The relationship between international trade and health increases as the flow of products, services, people, and investment rises, contributing to negative impacts on health. A country’s health system can be affected through the spread of communicable diseases across borders, the promotion of unhealthy products, and the flow of medical personnel.

In developing countries, this phenomenon is more obvious. For instance, TRIPS plus intellectual property protection may reduce access to affordable medicines or change the rules by which state-owned agencies operate, such as commercial entities, may affect local production [3].

International trade plays a crucial role in the Thai economy and the development of the country while also having a negative impact on the country’s health system.

Besides international trade that occurs through the market mechanism, Thailand has signed 14 free trade agreements. In contrast to the positive gains made from international trade, many people have expressed concerns regarding its negative impacts, particularly on people’s health and the health system of the country. For example, a recent study found that signing the Comprehensive and Progressive Agreement of Trans-Pacific Partnership (CPTPP) agreement would cost Thailand around THB 400 billion (USD 12.1 billion) more in drug expenses than it currently pays, while the country would also have to rely on drug imports for up to 89% of the products needed [4]. Thailand also made use of the flexibility allowed by the Trade-Related Aspects of Intellectual Property Rights (TRIPS) agreement—i.e., the implementation of the government use of license (GUL)—from 2007 to 2008 for the production and import of seven drugs used for the treatment of cancer, heart disease, and HIV, which could save the government budget of USD 566.5 million in five years. These savings were later spent on access to non-GUL essential medicines for other diseases and patients [5].

In addition, the trends surrounding new trade issues, such as those regulated under the CPTPP and related to wider aspects of health and the health system, are more complicated. It is therefore essential to determine what is already known and what gaps exist in order to plan for the future situation of trade and health in Thailand.

To the best of our knowledge, no scoping review on international trade and health in Thailand has yet been carried out. This scoping review (in which most research found focused on trade in the health service and access to medicine) aims to identify the existing knowledge on these issues and determine the knowledge gaps that need to be filled.

## 2. Methods

This study aims to report on the scope of available research literature concerning international trade and health in Thailand. The form of a scoping review was chosen by the research team following guidance from Grant and Booth (2009) [6].

We adopted the guidance for scoping reviews and followed the steps described by Arksey and O’Malley [7] as follows:

### 2.1. Specifying the Research Question

The question asked in this scoping review is, “How many studies on international trade and health in Thailand were carried out in the 30-year period between 1991 and 2020 and in which areas were they carried out?”

### 2.2. Identifying Relevant Studies

#### 2.2.1. Search Strategy and Sources

Unlike a systematic review, the objective of this review is to provide comprehensive evidence rather than analyze the outcomes of specific research questions.

In this study, international trade issues were defined in accordance with the trade topics under the World Trade Organization (WTO). This scoping review focuses on the following trade topics: (1) trade in services, (2) intellectual property, (3) trade in goods, (4) dispute settlement, and (5) cross-cutting issues. Health impacts refer to the effects on access to health services and the health of the population due to the direct and/or indirect consequences of international trade. The consequences also include the distribution of health products or health services as a result of international trade or changing import quotas for chemical substances used for the production of pharmaceutical supplies.

This review included peer-review studies, research reports, gray literature, theses, and dissertations published between 1991 and 2020 in either Thai or English language. Quantitative, qualitative, and mixed-methods studies were all included in this review. Studies that were not related to the content of international trade and health in the context of Thailand and publications in the forms of commentaries or editorials were excluded.

Literature search strategies were developed using medical subject headings (MeSH) and text words related to international trade and health in Thailand. The review methodology focused on databases to which the authors had access, such as PubMed, ISI Web of Science, Scopus, Networked Digital Library of Theses and Dissertations, the Conference Proceedings Citation Index, Thai Journals Online (ThaiJO), and Thai Digital Collection (TDC) databases. The search was performed between 1 July 2020 and 31 July 2020. Moreover, a manual search of relevant studies from the reference lists of the studies included from the primary search and the websites of related organizations was also conducted.

#### 2.2.2. Study Selection

**Figure 1:**
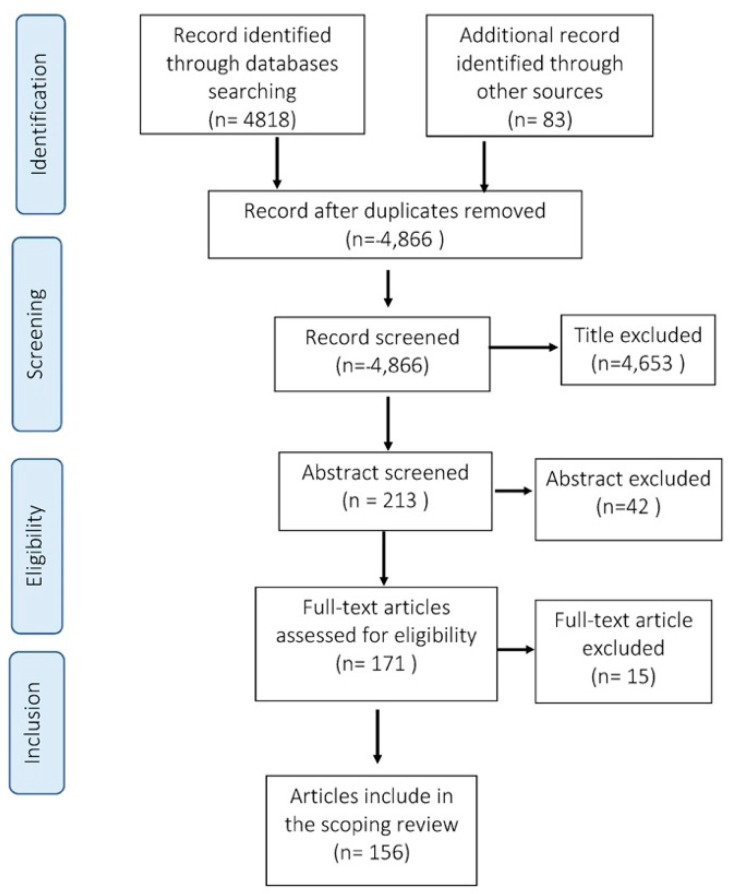
PRISMA flowchart shows the articles identified from the literature search from 1991 to 2020.

In the screening process, two researchers (KK and TB) selected studies to be included based on their titles and abstracts. All the included studies were compiled in the EndNote program. Full articles were screened against the inclusion and exclusion criteria. All screening and extraction procedures were completed in duplicate by KK and TB. Consultations with the project supervisors were arranged if any inconsistencies or disagreements regarding the included studies occurred between the researchers. Figure 1 summarizes the steps taken in the selection process according to the PRISMA statement [8].

### 2.3. Charting the Data

The data extraction table was developed based on the literature review and adjusted to provide a better extraction of information. An Excel program was used to create a table to extract information from the selected studies by six researchers, where studies with full text available were divided and assigned for recording. Each researcher independently extracted information and the project supervisors were available if any ambiguities in the data extraction occurred. After the extraction was completed, all data were combined and sent to the project supervisors for assessment. Study quality was not assessed during the scoping review, as the objective of this scoping review is to identify knowledge and gaps in the literature in order to suggest topics requiring further study in relation to international trade and health in Thailand [9,10].

### 2.4. Collating and Summarizing the Findings

The data were analyzed using frequencies and percentages for studies related to trade topics relating to the issues of international trade and health. Content analysis was employed to provide policy suggestions and recommendations for future studies on international trade and health in Thailand and identify research gaps needing further investigation.

## 3. Results

### 3.1. General Characteristics

**Figure 2:**
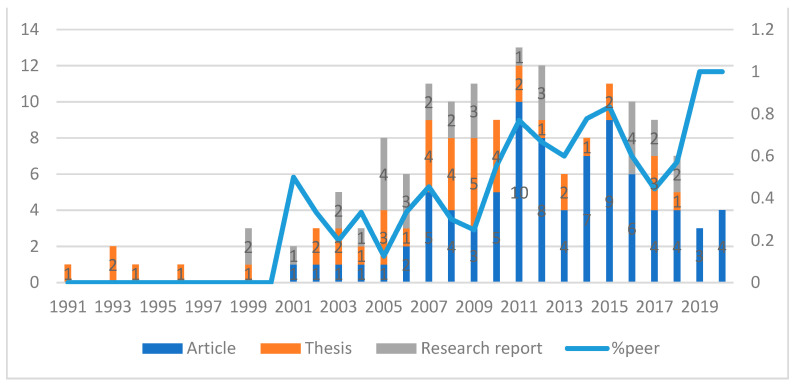
Overview of studies on international trade and health in Thailand.

We identified 156 studies carried out from 1991 to 2020 focusing on international trade and health in Thailand, including 82 articles, 43 theses, and 31 research reports. List of all articles included in the scoping review can be found in Supplementary Table S1. The majority of these studies were peer-reviewed articles, and their proportion tended to increase over time, while the number of theses decreased (Figure 2 illustrates the overview of studies on international trade and health in Thailand). This shows that the number of studies on international trade and health in Thailand started increasing from 2000, with the highest number of 13 studies, 10 peer-reviewed articles, 2 theses, and 1 research report being published in 2011. For all of the 156 studies, there were a total of 273 authors. However, 232 of them contributed to only one study. This could imply a lack of continuity in the research in this field.

**Figure 3:**
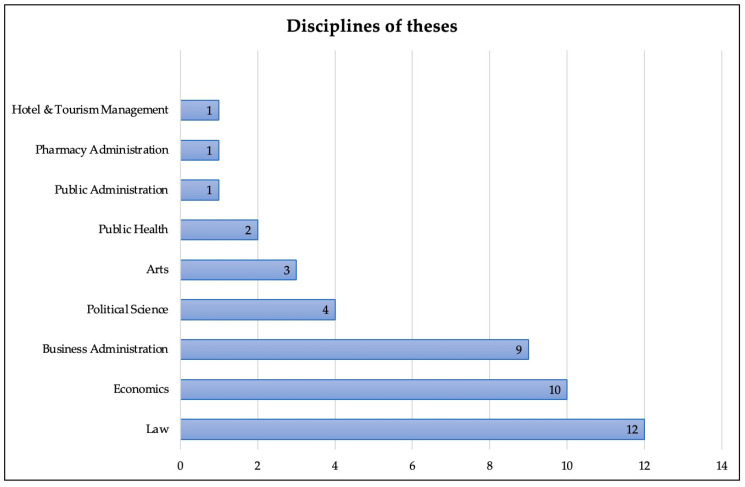
Disciplines of theses.

**Figure 4:**
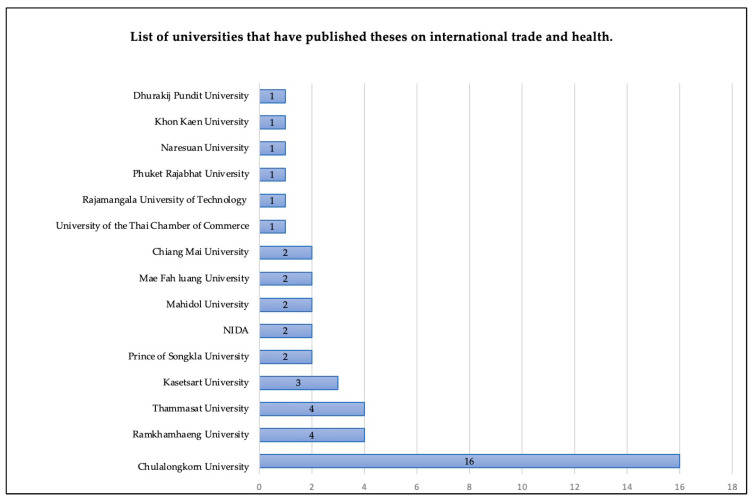
List of universities that have published theses on international trade and health.

For the theses, we found that the main type of theses produced were for the Master’s degree level. In total, there were 38 master theses and five doctoral theses. The most common fields of study were law (12 studies), followed by economics (10 studies) and business administration (9 studies). Chulalongkorn University published the highest number of studies. Details of the disciplines and universities are shown in Figure 3 and Figure 4, respectively.

**Figure 5:**
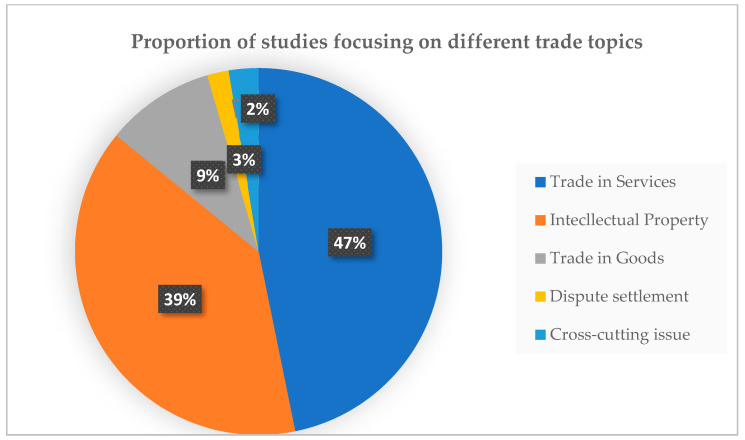
Proportion of studies focusing on different trade topics.

The proportion of studies focusing on different trade topics is shown in Figure 5. Most of the studies (47% or 73 studies) published focused on trade in services, followed by studies on intellectual property (39% or 61 studies). Sixteen studies (9%) focused on trade in goods. Three studies focused on dispute settlements. Another four studies investigated cross-cutting issues. The main focuses of the included studies are shown in Table 1.

### 3.2. Trade in Services and Health

The majority of the studies published on the topic of trade in health services focused on medical hub policy, although more than two thirds were on the positive impacts and benefits of medical tourism. As Thailand is one of the main medical destinations in Asia, with 3.42 million visits from medical tourists in 2018, it is not surprising that the majority of these studies focused on this topic. In addition, the medical hub policy in Thailand includes not only medical services but also wellness services. Thus, the medical hub policy is not only attractive for medical tourists, but also to scientists, physicians, researchers, and investors [11].

#### 3.2.1. Positive Impacts on Medical Hub Policy in Thailand

The majority of studies found described attempts to gain benefits from Thailand’s medical hub policy. Almost all of these studies focused on understanding the situations and factors that affected medical tourists’ decisions, which constitutes an important strategy for increasing the benefits of medical tourism in Thailand. These studies were divided into sub-issues with similar content in order to help us understand the needs of foreign tourists. However, all the studies differed from one another in specific details, such as the nationality of the tourists analyzed.

Studies on trade in health services could provide guidelines for improving the business sector in response to Thailand’s medical hub policy, as could studies on the needs and motivations of foreign tourists aiming to make use of Thailand’s medical tourism services [11,12,13].

Key findings from the studies in this category pointed out the opportunities for Thailand from its medical hub policy and medical tourism. Although Thailand has always been a tourist destination, there is room for improvement regarding meeting the needs of medical tourists as well as for the business sector and the country as a whole to gain more benefits, such as improving hospitals’ websites and engaging in proactive marketing [11,12,13].

#### 3.2.2. Negative Impacts on Medical Hub Policy in Thailand

Many studies analyzed the negative impacts of trade on public health and the health system. Most of these focused on the negative effects on medical personnel, especially the internal brain drain of medical staff to larger hospitals receiving foreign patients [14,15].

As a result of the economic crisis that occurred in 1997, foreign investors began to invest in private hospitals with a very low bed occupancy from 2001, hence accelerating the rise of private hospitals. The returns of this economic growth, in turn, have created another cycle of internal brain drain [16].

### 3.3. Intellectual Property System and Health

This scoping review found 61 studies related to the effects of the intellectual property protection of health products, especially drugs and medical supplies. These topics found three main focuses: (1) the TRIPS agreement and negative impacts, (2) compulsory licenses (CLs), and (3) the analysis of the Thai Patent Act.

A study focusing on the analysis of Thailand’s Patent Act (B.E.2535) revealed negative impacts on public access to medicines due to the provisions of the law in anticipation of price movements or low-grade technology transfer; this study proposes that some aspects of the existing Patent Act should be changed, such as the term or duration of patent protection, non-patentable subject matter, the rights and privileges of patentees, and import monopolies [17]. Patenting does increase price and challenges access to medicine and technologies [18] and increases the costs of producing drugs for local Thai pharmaceutical companies [19].

A number of studies focused on demonstrating the degree of impact on drug access from IP systems—in particular, drug patents and the evaluation of the patent drug market monopoly. One study found that Thailand had a high incidence of HIV/AIDS infection but that most patients lacked access to treatment that could greatly improve their lives as a result of the high cost of essential drugs [20].

One key solution is to use TRIPS flexibility to address the drug access situation. This review found 21 studies focused on compulsory licenses (CLs) and drug access. The most common issue was the analysis of the experience in issuing the use of CL on seven drugs from 2006 to 2008 in Thailand [21]. The studies in this group evaluated the worth of issuing a CL and discussed the historical lesson learned from the use of CL. The results of a study entitled “Government use licenses in Thailand: An assessment of the health and economic impacts” showed that overall Thai exports have increased, despite Thailand withdrawing from GSP 3: a list of items exported to the US. In addition, there was no correlation between the use of CL and the level of inward foreign investment from 2002 to 2008 [21]. The studies related to the intellectual property and health topic show that the use of CL in Thailand is worthwhile, but its operation may be challenging. There are many sensitive issues that have to be taken into account, including the readiness of the organization and political and civil society sectors [22]. All of these components are extremely important.

However, this scoping review found both positive and negative effects of Free Trade Agreements (FTAs) related to intellectual property. On the positive side, domestic drug manufacturers would have the opportunity to improve their quality and medicines could be exported to countries with FTAs more conveniently [23]. For medicine, there was a trend of increase in the volume and import–export value [24].

On the negative side, an increase in drug costs could be caused by TRIPS-Plus, which is usually mentioned in the new proposal of FTA. For example, the Thai–US FTA required restrictions on the grounds of compulsory licensing, the expansion of the patent scope, and limits to challenging potentially invalid patents. This requirement might further limit the use of important existing flexibilities in drug patents [25].

Linking to the free trade agreements, the patent has the most impact on the research and development of the pharmaceutical industry. Moreover, this study found that Thailand has limited capacity to compete in the world market [26].

### 3.4. Other Related Studies and Cross-Cutting Issues

As stated earlier, this scoping review focuses on five main trade topics as per the WTO’s classification. However, it found that the number of studies on trade in goods and dispute settlement was limited. For trade in goods, most studies focus on the policy analysis of products that are harmful to health, such as alcohol and tobacco. Many studies provide evidence to support government policy and to ensure that these policies are not against trade rules and agreements [27].

Similarly, there were only a few studies on dispute settlements. There was one study focusing on an analysis of measures taken to reduce tobacco consumption due to public health concerns [28] and another on the readiness of Thailand’s law to comply with the World Health Organization’s Framework Convention on Tobacco Control (FCTC) [29]. Moreover, another relevant study was also found that focused on dispute settlements and the environment in a case study of gold mining in Thailand [30].

There were only three studies classified as focusing on cross cutting issues that overlap with many topics. The first study focused on policy coherence between health-related trade and health system development in Thailand [31]. The second study was “Capacity building for global health diplomacy: Thailand’s experience of trade and health”. This paper discussed Thailand’s experience in trade and health [32]. The last study was “Trade and health diagnostic tool: the Thailand perspective”, which discussed the impact of trade on four aspects relating to the health of the Thai population, namely: (i) macroeconomy and trade; (ii) trade in health goods; (iii) trade in health bad or products not conducive to health; and (iv) trade in health services [33].

## 4. Discussion

### 4.1. Scoping Review as a Chosen Method for This Study

Systematic reviews only cover peer-reviewed papers but not gray literature and literature not published in searchable databases. A large proportion of works on international trade and health in Thailand are not published in peer-reviewed journals; however, many of these are reports prepared for relevant agencies as part of documents providing decision-making support. In addition, the timeline and funding of the project did not allow for the quality assessment that is required for systematic reviews. Hence, the format of a scoping review was chosen for this study.

### 4.2. Direct and Indirect Association between International Trade and the Positive and Negative Effects on Health

The effects of increased international trade on health are both direct and indirect [34]. The positive direct effect of international trade is an increased availability of goods that are essential for health, such as pharmaceuticals, medicines, and food. On the negative side, international trade increases the availability of goods that are harmful to health, such as alcohol and tobacco. It also leads to the migration of health professionals and the potential spread of infectious diseases. The indirect effect of international trade was found to contribute to poverty reduction, the stimulation of development, better living conditions, and greater knowledge dissemination. However, the negative indirect effects include increased use of vehicles for transportation; greater use of fossil fuels; greater levels of pollution; and worsened effects of climate change, including droughts, floods, and rising sea levels [35,36,37,38].

This scoping review supports the idea that international trade is associated with both positive and negative effects on health, either directly or indirectly. However, this review finds that studies in Thailand lack a balanced view of both the positive and negative effects. Studies on the negative effects of trade are found more commonly than studies on the positive effects. This might be because the negative health impacts of international trade are more alarming and thus gain more attention; most studies therefore concern the negative health impacts of trade.

### 4.3. Policy-Related Studies

A number of studies related to important policies are being discussed at the time of their publication. The studies on trade in health services found in this study were related to Thailand’s medical hub policy. The medical hub policy in Thailand was established in 2003. Studies on Thailand’s medical hub policy have been conducted since 2003; they have focused on both the positive and negative impacts on health and are still currently being conducted. Similarly, since the revision of the Patent Act in 1999, a number of studies focusing on the impacts on health caused by the Patent Act were also found.

### 4.4. Research Gaps

As shown in Table 1, 73 out of 156 studies considered in this scoping review were related to trade in services. Of these, 52 studies focused on mode 2 consumption abroad, analyzing both the negative and positive impacts of the medical hub policy in Thailand. However, we found a smaller number of studies on other modes of services, while none were found on mode 1 cross border supply.

Out of the 61 studies found on intellectual property issues, these mainly discussed the negative impacts of the TRIPS agreement on health and the issue of compulsory licensing (CL) (as Thailand has issued CL for eight drugs to increase access and improve lives). However, the review did not find any studies on the positive side of intellectual property rights or how the intellectual property system can promote innovations in health in Thailand.

On trade in goods, most studies found focused on trade and health policies, especially health prevention policies, such as plain packaging for tobacco and alcoholic beverages. In turn, health policies would impact trade through technical barriers to trade. This review also found a limited number of studies on the pharmaceutical industry, medical devices, and other health products. As the situation of international trade and particularly that for products and intellectual properties is very dynamic and becoming more complicated, empirical evidence supporting policy decisions and the evaluation of trade and health policies as well as their impacts is necessary.

Policy coherence, evidence, and participation are recommended in order to build up ‘healthy trade’ policies. It was suggested that empirical evidence is an important step towards reaching a more sustainable form of trade liberalization [39]. This scoping review found only three studies focusing on policy coherence. Therefore, this review recommends that further studies should be carried out to provide additional mechanisms for creating policy coherence between trade and the health sector and more critical evidence on trade and health issues, particularly regarding measuring, monitoring, and evaluating the impact of international trade on health. Multidisciplinary approaches and multi-sector collaboration will also be required.

One observation of this review is that many studies have not been published and are not available on databases. This scoping review covered seven databases, both international and domestic; however, many important studies were found from our manual search process on the websites of relevant agencies. Therefore, relevant organizations who generate knowledge or information should make them accessible. Making research articles available on international databases will be another way to increase the visibility of international trade and health issues.

It is very important that knowledge on international trade and health be understandable to the public. This scoping review recommends that further studies should be carried out to provide more critical evidence on trade and health issues. In particular, the measuring, monitoring, and evaluation of the impact of international trade on health are important. Multidisciplinary approaches and multi-sector collaboration should be included [39].

### 4.5. Limitation of the Study

This study has a few limitations. First, the scope of this study focused on the main trade topics as per the World Trade Organization’s classification. In accordance with the WTO’s website, trade topics were classified into many different topics and subtopics, such as goods, services, intellectual property, dispute settlements, regional trade agreements, Doha development agenda, building trade capacity, and trade monitoring. However, this study scoped and analyzed previous studies on international trade and health in Thailand, where researchers selected topics under the main trade topics mentioned above as the framework of the study, and this also linked to the search terms used in this study. This scoping review and the search terms used may not be sufficiently extensive. Therefore, this study may not be able to compile all relevant studies on international trade and health in Thailand. Second, since the search process used was able to find many studies despite the research period of this scoping review being limited, the researchers applied a title screening process as the first step in order to screen out any unrelated papers. It is possible that some relevant studies were excluded if their titles did not reflect the main focus of the study.

## 5. Conclusions

This scoping review is the first review of studies on international trade and health in Thailand during the 30-year period from 1991 to 2020. This review found a total of 156 studies: 82 academic articles, 43 theses and dissertations, and 31 research reports. Studies on the trade in services were the most prominent, with 73 in total, accounting for 47% of all the studies. These were followed by 61 studies on intellectual property, accounting for 39% of all the studies. However, the number of studies on trade in goods, dispute settlement mechanisms, and cross-cutting issues was very limited. This study also found a limited number of studies on trade in health services focusing on mode 1, cross border supply; mode 3, commercial presence; and mode 4, presence of natural persons. There were also a limited number of studies on trade in health-related goods in terms of the analysis of the positive and negative impacts of international trade in Thailand on aspects such as the pharmaceutical industry and medical equipment. Additionally, no studies with a positive view of intellectual property rights in Thailand were found. This review reveals that there should be more studies focusing on issues relating to the impacts of trade on health-related goods in terms of imports and exports and the industry analysis of the positive and negative impacts of international trade in Thailand, such as the pharmaceutical industry and medical equipment. In the words of Grant and Booth (2009), “scoping review cannot usually be regarded as a final output in their own right”; further reviews, such as systematic reviews, are needed in order to identify the existence of knowledge and knowledge gaps. In addition, knowledge management concerning international trade and health matters is encouraged in order to provide a better understanding of their correlations and predict their potential future impacts. This constitutes an important contribution to the implementation of international trade policy that has both positive and negative impacts on public health and the health system.

## Figures and Tables

**Table 1:** Numerical distribution by trade topic and the main focus of the included studies.

Trade Topics	Main Focus	Number	Percentage
Trade in services	Mode 1: Cross border supply	0	0
	Mode 2: Consumption abroad: positive impacts on medical hub policy in Thailand	37	24
	Mode 2: Consumption abroad: negative impacts on medical hub policy in Thailand	15	10
	Mode 3: Commercial presence	1	1
	Mode 4: Presence of natural persons	10	6
	Focus on more than 1 mode	10	6
Intellectual property	TRIPS agreement and negative impact	27	17
	Compulsory license (CL)	21	13
	Analysis of Thai Patent Act	13	8
Trade in goods	Healthy products	4	3
	Unhealthy products	11	7
Dispute settlement	Environment	1	1
	Unhealthy products	2	1
Cross-cutting issue	Impact of FTAs	1	1
	Policy coherence	3	2
**Total**		**156**	**100**

## Data Availability

No new data were created or analyzed in this study. Data sharing is not applicable to this article.

## Supplementary Materials

The following are available online at https://www.mdpi.com/article/10.3390/ijerph182111692/s1. Table S1: List of all articles included in the scoping review.
