# Supplementary material for: International Trade and Health in Thailand: A Scoping Review
Source: Int J Environ Res Public Health. 2021 Nov 7;18(21):11692. doi: 10.3390/ijerph182111692 (PMC8583137; doi:10.3390/ijerph182111692)
Supplement: Supplementary file 1 [file ijerph-18-11692-s001.zip › ijerph-1418421-supplementary.pdf]

**Table S1.** List of all articles included in the scoping review.

| **All articles include in the scoping review** | | | |
|---|---|---|---|
| **No.** | **Document Reference (APA)** | **Trade topics** | **Document Type** |
| 1 | Aimsakul, A. (2010). Free trade agreement in health services: A case study on the effects and problems of the free trade agreement for dental practitioners. Eau Heritage Journal, 4(1), 69-79. | Trade in services | Peer-reviewed |
| 2 | Aizura, A. (2010). Feminine transformations gender reassignment surgical tourism in Thailand. Medical Anthropology, 29(4), 424-443. https://doi.org/10.1080/01459740.2010.501314 | Trade in services | Peer reviewed |
| 3 | Akaleephan, C. (2011). Identifying national priority areas under WHA61.21 by ascertaining status of pharmaceuticals, herbal medicines, vaccines and medical technologies in Thailand (Research report). Bangkok: International Health Policy Program. | Intellectual property | Research report |
| 4 | Akaleephan, C., Akaleephan, C., Kessomboon, N., Kijtiwatchakul, K., Eksaengsri, A., & Limpananont, J. (2020). International trade agreement and impact on the medicine system : Causal Relations?. Journal Of Health Science, 29(January - February), S153-S166. | Intellectual property | Peer reviewed |
| 5 | Akaleephan, C., Tangcharoensathien, V., Eksaengsri, A., Wibulpolprasert, S., Sakulbumrungsil, R., & Udomaksorn, S. et al. (2005). Impact and guideline for negotiation on the bilateral agreement: Estimation of the impact of intellectual property right on price and access to pharmaceutical. (Research report). Bangkok: International Health Policy Program. | Intellectual property | Research report |
| 6 | Akaleephan, C., Wibulpolprasert, S., Sakulbumrungsil, R., Luangruangrong, P., Jitraknathee, A., & Aeksaengsri, A. et al. (2009). Extension of market exclusivity and its impact on the accessibility to essential medicines, and drug expense in Thailand: Analysis of the effect of TRIPs-Plus proposal. Health Policy, 91(2), 174-182. https://doi.org/10.1016/j.healthpol.2008.12.009 | Intellectual property | Peer-reviewed |
| 7 | Alberti, F., Giusti, J., & Papa, F. (2014). Competitiveness policies for medical tourism clusters: Government initiatives in Thailand. International Journal Of Economic Policy In Emerging Economies, 7(3), 281-309. https://doi.org/10.1504/IJEPEE.2014.065252 | Trade in services | Peer-reviewed |
| 8 | Artsanthia, J. & Chaleoykitti, S., 2016. ASEAN Community and Thai health system. Journal of The Royal Thai Army Nurses, 17(3), pp.10-16. | Trade in services | Peer-reviewed |
| 9 | Arunanondchai, J., Pachanee, C., & Akaleephan, C. (2007). Trade and health diagnostic tool: The Thailand perspective (Research report). Bangkok: International Health Policy Program. | Cross-cutting issue | Research report |
| 10 | Baiprao, C. (1999). The analysis of Economics factors' impact on imported drugs consumption in Thailand during 1992-1997 (Independent Study). Chiang Mai University. | Trade in goods | Independent Study |
| 11 | Bate, R. (2007). Thailand and the drug patent wars. American Enterprise Institute For Public Policy Research. | Intellectual property | Peer reviewed |
| 12 | Bochaton, A. (2015). Cross border mobility and social networks : Laotians seeking medical treatment along the Thai border. Social Science & Medicine, 124, 364-373. https://doi.org/10.1016/j.socscimed.2014.10.022 | Trade in services | Peer reviewed |
| 13 | Borisuthri, P. (2010). Protection of local wisdom relating to traditional medicine under TRIPs Agreement (Master's thesis). Thammasat University. | Intellectual property | Thesis |
| 14 | Carreon, J., Todd, R., & Knox, J. (2011). Medical tourism communication of a Thai private hospital website. Journal Of Applied Linguistics And Professional Practice, 8(2), 165-185. https://doi.org/10.1558/japl.v8i2.165 | Trade in services | Peer reviewed |

| | | | |
|---|---|---|---|
| 15 | Center of Excellence on Hazardous Substance Management. (2018). Empirical data on import and management of electronic waste and sludge in Thailand (Phase one) (Research report). Bangkok: International Trade and Health. | Trade in goods | Research report |
| 16 | Chaipinit, C. (2010). The political economy of the intellectual property rights policy: A case study of compulsory licensing in Thailand (Doctoral dissertation). Chulalongkorn University. | Intellectual property | Dissertation |
| 17 | Chaiyasong, S., Markchang, K., & Jaichuen, N. (2016). Literature review on the impact of Trans-Pacific Partnership Agreement on public health and health systems in Thailand: focusing on alcohol and tobacco. (Research report). Bangkok: International Health Policy Program. | Trade in goods | Research report |
| 18 | Changsalak, P. (2002). New economic order / new trading order "Agreement on trade-related aspects of intellectual property rights": Its impacts on Thailand (Master's thesis). Ramkhamhaeng University. | Intellectual property | Thesis |
| 19 | Chantanavanich, S. (2009). The effect of the European Union's patients' rights in cross-border healthcare initiative on medical tourism in Thailand (Master's thesis). Chulalongkorn University. | Trade in services | Thesis |
| 20 | Chardsumon Prutipinyo and Nithat Sirichotiratana.(2015) Protection of alcohol control policies from the influence of free trade agreement. Public Health & Health Laws Journal, 2, 119-134. | Trade in goods | Peer reviewed |
| 21 | Chompoopetch, W. (1994). Trade problems between Thailand and The United State : A case study of pharmaceutical patent (Master's thesis). Ramkhamhaeng University. | Trade in goods | Thesis |
| 22 | Chongthanavanit, P., & Kheokao, J. (2018). Factors influencing the use of dental services by foreign tourists in Thailand. Asian Journal For Public Opinion Research, 5(2), 63-83. http://dx.doi.org/10.15206/ajpor.2018.5.2.63 | Trade in services | Peer-reviewed |
| 23 | Chotesungnoen, K. (2007). Politics and bureaucracy behind Thai government's issuance of compulsory licensing (CL) (Master's thesis). Chulalongkorn University. | Intellectual property | Thesis |
| 24 | Cohen, E. (2020). Medical tourism in Thailand. Au Gsb E Journal, 24-37. | Trade in services | Peer reviewed |
| 25 | Dhanarajan, S. (2001). The impact of patent rules on the treatment of HIV/AIDS in Thailand (Research report). Oxfam. | Intellectual property | Research report |
| 26 | Fathelrahman, A., Li, L., Borland, R., Yong, H., Omar, M., & Awang, R. et al. (2013). Stronger pack warnings predict quitting more than weaker ones: Finding from the ITC Malaysia and Thailand surveys. Tobacco Induced Diseases, 11(20). https://doi.org/10.1186/1617-9625-11-20 | Trade in goods | Peer reviewed |
| 27 | Ford, N., Wilson, D., Chaves, G., Lotrowska, M., & Kijtiwatchakul, K. (2007). Sustaining access to antiretroviral therapy in the less-developed world: Lessons from Brazil and Thailand. AIDS, 21(4), S21-S29. https://doi.org/10.1097/01.aids.0000279703.78685.a6. | Intellectual property | Peer-reviewed |
| 28 | Wangchuka, K.,& Supanatsetakul,N. (2015). Foreign medical practitioners: requirements for medical practice and postgraduate training in Thailand under ASEAN Economic Community liberalization in 2015. Asian Biomedicine, 9(6),777-782. | Trade in services | Peer-reviewed |
| 29 | Forman, L. (2012). From Trips-Plus to Rights-Plus? Exploring right to health impact assessment of trade-related intellectual property rights through the Thai experience. Asian Journal Of WTO & International Health Law And Policy, 7(2), 347-375. | Intellectual property | Peer-reviewed |

| | | | |
|---|---|---|---|
| 30 | Fukahori, H., Baba, Y., Hioki, F., Monkong, S., Intarasombat, P., & Malathum, P. (2011). Healthcare services for Japanese elderly long-staying in Thailand from the perspective of the patient and healthcare providers: A survey study. Arch Gerontol Geriatr, 53(2), 168-173. https://doi.org/10.1016/j.archger.2010.08.007 | Trade in services | Peer reviewed |
| 31 | Guennif, S. (2017). Evaluating the usefulness of compulsory licensing in developing countries: A comparative study of Thai and Brazilian experiences regarding access to aids treatments. Developing World Bioethics, 17(7), 90-99. https://doi.org/10.1111/dewb.12124. | Intellectual property | Peer-reviewed |
| 32 | Guennif, S., & Mfuka, C. (2003). Impact of intellectual Property rights on Aids public health policy in Thailand, 137-150. | Intellectual property | Peer reviewed |
| 33 | Hadsarang, N. (2008). Thailand's patent protection and access to medicine in the framework of trade-related aspects of intellectual property right (Master's thesis). Thammasat University. | Intellectual property | Thesis |
| 34 | Harryono, M., Huang, Y., Miyazawa, K., & Sethaput, V. (2006). Thailand medical tourism cluster (Research report). Harvard Business School. | Trade in services | Research report |
| 35 | Havarungsi, N. (2006). The competitive advantage of Thai medical tourism industry (Independent study). Kasetsart University. | Trade in services | Independent study |
| 36 | Hirankitti, P., Meejinda,P., , Hirankitti, S., Manjing, S.,& Sayapan,U.(2009). A Study of Medical Tourism Behavior of Foreign Tourists (Research report). Bangkok: Rajamangala University of Technology Thanyaburi. | Trade in services | Research report |
| 37 | Huang, Y. (2007). Negotiating health in Thailand: AIDS, global patent regime, and health social movement. | Intellectual property | Peer reviewed |
| 38 | Jaisa-ard, R. (2004). Patient rights protection by using compulsory licensing : A case study for accessing to Efavirenz in access to care program (Master's thesis). Mahidol University. | Intellectual property | Thesis |
| 39 | Jaiua, M. (2006). Strategic planning for medical tourism development for international tourists: A case study of Bangkok (Master's thesis). Naresuan University. | Trade in services | Thesis |
| 40 | James, P. (2012). The impact of medical tourism on Thai private hospital management: informing hospital policy. Global Journal Of Health Science, 4(1), 127-139. https://doi.org/10.5539/gjhs.v4n1p127 | Trade in services | Peer-reviewed |
| 41 | James, P. (2020). Services marketing and medical tourism: The impact on private health services in Bangkok. Journal Of Management Research, 12(1), 37-56. https://doi.org/10.5296/jmr.v12i1.15819 | Trade in services | Peer reviewed |
| 42 | Janjaroen, W., & Supakankunti, S. (2000). International trade in health services in the millennium: The case of Thailand, 87-106. | Trade in services | Peer reviewed |
| 43 | Jencharoenwong, S., & Assenov, I. (2010). Medical tourism strategies for Thailand. The 8Th Apaccherie Conference 2010. | Trade in services | Peer reviewed |
| 44 | Jensen, H., Keogh-Brown, M., Shankar, B., Aekplakorn, W., Basu, S., & Cuevas, S. et al. (2019). International trade, dietary change, and cardiovascular disease health outcomes: Import tariff reform using an integrated macroeconomic, environmental and health modelling framework for Thailand. SSM - Population Health, 9, 1-15. https://doi.org/10.1016/j.ssmph.2019.100435 | Trade in goods | Peer-reviewed |
| 45 | Jindaratthanaporn, N., Markchang, K., Nasurb, K., & Chaiyasong, S. (2017). A Review of Alcohol Control and Regulations for the Excise Tariff subordinate legislation B.E.2560 (2017): A Case study with beverages containing not more than 0.5% alcohol by volume (Research report). Bangkok: Center of Alcohol Studies. | Trade in goods | Research report |

| | | | |
|---|---|---|---|
| 46 | Kanchanachitra, C., Lindelow, M., Johnston, T., Hanvoravongchai, P., Lorenzo, F., & Huong, N. et al. (2011). Human resources for health in southeast Asia: Shortages, distributional challenges, and international trade in health services. Lancet, 377(9767), 769-781. https://doi.org/10.1016/S0140-6736(10)62035-1 | Trade in services | Peer-reviewed |
| 47 | Kanchanachitra, C., Tiewkul, W., Pachanee, C., & Tangcharoensathien, V. (2004). A situational review and knowledge to develop a series of research projects on trade in health-related services (Research report). Bangkok: Thailand Research Fund. | Trade in services | Research report |
| 48 | Karnda, S. (2008). The development of the marketing mix factors for foreign patients of private hospitals: A case study in Phuket (Master's thesis). Phuket Rajabhat University. | Trade in services | Thesis |
| 49 | Kessomboon, N., Jaisa-Ard, R., Sermsinsiri, V., Chanthapasa, K., & Kanjanarach, T. (2002). Methodological development for health impact assessment: A case study from TRIPs agreement (Research report). Bangkok: Health Systems Research Institute. | Intellectual property | Research report |
| 50 | Kessomboon, N., Limpananont, J., Kulsomboon, V., Maleewong, U., Eksaengsri, A., & Paothong, P. (2010). Impact on access to medicines from TRIPS-Plus: A case study of Thai-US FTA. The Southeast Asian Journal Of Tropical Medicine And Public Health, 41(3), 667-677. | Intellectual property | Peer-reviewed |
| 51 | Khaofong, N. (2018). Expectation and perception of border healthcare in Chiang Rai province, Thailand (Master's thesis). Mae Fah Luang University. | Trade in services | Thesis |
| 52 | Kittitrakul, C., Lawpoolsri, S., Kusolsuk, T., Olanwijitwong, J., Tangkanakul, W., & Piyaphanee, W. (2015). Travelers' diarrhea in foreign ravelers in southeast Asia: A cross-sectional study in Bangkok, Thailand. Am J Trop Med Hyg, 93(3), 485-490. https://doi.org/10.4269/ajtmh.15-0157 | Trade in services | Peer reviewed |
| 53 | Klongdee, S. (2011). Factors determining the demand and competitiveness of medical tourism in Thailand (Master's thesis). Chulalongkorn University. | Trade in services | Thesis |
| 54 | Kogiso, K. (2012). Thai massage and health tourism in Thailand: Tourism acculturation process of "Thai Massage". International Journal Of Sport And Health Science, 10, 65-70. https://doi.org/10.5432/ijshs.201209 | Trade in services | Peer reviewed |
| 55 | Komolsevin, R., & Khasuwan, S. (2016). Medical-related factors and perceived image of medical tourism in Thailand: Case study of meddle-east tourists. Sakon Nakhon Graduate Studies Journal, 61(13), 49-58. | Trade in services | Peer-reviewed |
| 56 | Kuanpoth, J. (2005). Patents and access to medicines in Thailand – the ddI case and beyond. Intellectual Property Quarterly, 18-40. | Intellectual property | Peer reviewed |
| 57 | Kuanpoth, J. (2006). TRIPS-Plus intellectual property rules: Impact on Thailand's public health. The Journal Of World Intellectual Property, 9(5), 573–591. https://doi.org/10.1111/j.1422-2213.2006.00303.x | Intellectual property | Peer reviewed |
| 58 | Kuanpoth, J. (2008). Appropriate patent rules in developing countries - Some deliberations based on Thai legislation. Journal Of Intellectual Property Rights, 13(5), 447-455. | Intellectual property | Peer reviewed |
| 59 | Kuanpoth, J., Kripke, G., & Weinberg, S. (2006). Public health at risk a US Free Trade Agreement could threaten access to medicines in Thailand (Research report). UK: Oxfam International. | Intellectual property | Research report |
| 60 | Kuek, V., Phillips, K., & Kohler, J. C. (2011). Access to medicines and domestic compulsory licensing: learning from Canada and Thailand. *Global public health*, *6*(2), 111–124. https://doi.org/10.1080/17441690903575255 | Intellectual property | Peer-reviewed |

| | | | |
|---|---|---|---|
| 61 | Kyung-Bok Son, Chang-yup Kim & Tae-Jin Lee (2019). Understanding of for whom, under what conditions and how the compulsory licensing of pharmaceuticals works in Brazil and Thailand: A realist synthesis. (2019), 14(1), 122-134. https://doi.org/10.1080/17441692.2018.1471613 | Intellectual property | Peer reviewed |
| 62 | Landreville, P. (2016). Middle Eastern consumers' perceptions towards medical tourism in Thailand. The Bangkok Medical Journal, 12, 39-51. | Trade in services | Peer-reviewed |
| 63 | Lertsuphajirachote, C. (2005). The behavior of foreign tourist to select hospital in Chnagwat Phuket (Master's thesis). Chulalongkorn University. | Trade in services | Thesis |
| 64 | Li, L., Borland, R., Yong, H., Sirirassamee, B., Hamann, S., Omar, M., & Quah, A. (2015). Impact of Point-of-Sale tobacco display bans in Thailand: Findings from the International Tobacco Control (ITC) Southeast Asia Survey. Int J Environ Res Public Health, 12(8), 9508–9522. https://doi.org/10.3390/ijerph120809508 | Trade in goods | Peer reviewed |
| 65 | Limstit, P. (2013). Mechanisms to prevent the interference with tobacco control of the tobacco industry under Article 5.3 of the WHO framework convention on tobacco control in Thailand. Journal Of Health Systems Research, 7(1), 126-136. | Trade in goods | Peer reviewed |
| 66 | Liu, Z. (2017). Chinese tourists perception for medical tourism services in Bangkok, Thailand (Master's thesis). Chulalongkorn University. | Trade in services | Thesis |
| 67 | Lybecker, K., & Fowler, E. (2009). Compulsory licensing in Canada and Thailand: Comparing regimes to ensure legitimate use of the WTO rules. Journal Of Law, Medicine & Ethics, 37(2), 222-239. https://doi.org/10.1111/j.1748-720X.2009.00367.x | Intellectual property | Peer-reviewed |
| 68 | MacKenzie, R. (2012). Trade policy, not morals or health policy': The US Trade Representative, tobacco companies and market liberalization in Thailand. Glob Soc Policy, 12(2), 149-172. https://doi.org/10.1177/1468018112443686 | Trade in goods | Peer-reviewed |
| 69 | MacNaughton, G., & Forman, L. (2015). Human rights and health impact assessments of trade-related intellectual property rights: A comparative study of experiences in Thailand and Peru. Journal of Human Rights, 14(1), 124-148. https://doi.org/10.1080/14754835.2014.987738 | Intellectual property | Peer-reviewed |
| 70 | Makmork, V. (2009). Thailand's positions with regards to health service sector under the draft Thai-US FTA (Master's thesis). Chulalongkorn University. | Trade in services | Thesis |
| 71 | Maleewong, U., Kessomboo,N., Eksangsri, A., Asawintarangkul,S.,& Kittivejakul,(2012). Evergreening drug patents in Thailand and the impact estimation. (Research report). Bangkok: Health Systems Research Institute. | Intellectual property | Research report |
| 72 | Maluangnon, K. (2003). Compulsory licensing of antietroviral: Policy options (Doctoral dissertation). Mahidol University. | Intellectual property | Thesis |
| 73 | Mansanguan, C., Matsee, W., Petchprapakorn, P., Kuhakasemsin, N., Chinnarat, N., Olanwijitwong, J., & Piyaphanee, W. (2016). Health problems and health care seeking behavior among adult backpackers while traveling in Thailand. Tropical Diseases Travel Medicine And Vaccines, 2(9). https://doi.org/10.1186/s40794-016-0026-9 | Trade in services | Peer-reviewed |
| 74 | Maturodprichakul, M. and Jankandee, W. (2012). Legal problems of implementing the Doha declaration on the TRIPs agreement and public health: Case study of AIDs drug patent in Thailand (Research report). Bangkok: Mae Fah Luang University. | Intellectual property | Research report |
| 75 | Maung, N., & Walsh, J. (2014). Decision factors in medical tourism: Evidence from Burmese visitors to a hospital in Bangkok. Journal Of Economics And Behavioral Studies, 6(2), 84-94. https://doi.org/10.22610/jebs.v6i2.472 | Trade in services | Peer reviewed |

| | | | |
|---|---|---|---|
| 76 | Mayakul, T., Mayakul, S., & Prasad, R. (2018). A sustainable medical tourism framework based on the enterprise architecture design: The case in Thailand. Journal Of Green Engineering, 8(3), 359-388. https://doi.org/10.13052/jge1904-4720.838 | Trade in services | Peer-reviewed |
| 77 | Mekritthikrai, V. (2008). Attitude of officials who work in health organziations in Bangkok towards Thailand the medical hub of Asia: A case study of effects on Thai health system (Master's thesis). Thammasat University. | Trade in services | Thesis |
| 78 | Mohara, A., Yamabhai, I., Chaisiri, K., Tantivess, S., & Teerawattananon, Y. (2012). Impact of the introduction of government use licenses on the drug expenditure on seven medicines in Thailand. Value In Health, 15(1), S95-S99. https://doi.org/10.1016/j.jval.2011.11.016 | Intellectual property | Peer-reviewed |
| 79 | Mongkonporn, W., Akaleephan, C., Kanchanachitra, C., & Tangcharoensathien, V. (2003). Supply and demand for medical services for foreign patients: impacts on health systems and health workers in Thailand.(Research report). Bangkok: International Health Policy Program. | Trade in services | Research report |
| 80 | Mungmunpuntipantip, R. (2019). Public health-oriented Intellectual Property and Legal Issue, View from Thai Context. Journal Of Intellectual Property Rights Law, 2(1), 9-12. | Intellectual property | Peer reviewed |
| 81 | Na Ranong, A., Na Ranong, V., & Jindarak, S. (2009). A development guideline for Thailand's medical Hub (Research report). Bangkok: National Institute of Development Administration. | Trade in services | Research report |
| 82 | NaRanonga, A., & NaRanong, V. (2011). The effects of medical tourism: Thailand's experience. Bull World Health Organ, 89(5), 336–344. https://doi.org/10.2471/BLT.09.072249 | Trade in services | Peer-reviewed |
| 83 | Niyomrai, N. (2002). Measures to prevent effects on patents to medicines (Master's thesis). Ramkhamhaeng University. | Intellectual property | Thesis |
| 84 | Nooseisai, M., Wang, Y., Hongsranagon, P., & Munisamy, M. (2016). Medical tourism within the medical hub policy: Reviewing the need of a balanced strategy for health inequality reduction in a Thai context. J Health Res., 30(6), 445-450. https://doi.org/10.14456/jhr.2016.58 | Trade in services | Peer-reviewed |
| 85 | Noppharesksawat, K. (2018). Legal measures on controlling medical tourism facilitator. Thammasat Business Law Journal, 6. | Trade in services | Peer-reviewed |
| 86 | Noree, T., Hanefeld, J., & Smith, R. (2014). UK medical tourists in Thailand: They are not who you think they are. Globalization And Health, 10(29), 1-7. https://doi.org/10.1186/1744-8603-10-29 | Trade in services | Peer reviewed |
| 87 | Noree, T., Hanefeld, J., & Smith, R. (2016). Medical tourism in Thailand: a cross-sectional study. Bull World Health Organ, 94(1), 30-36. https://doi.org/10.2471/BLT.14.152165 | Trade in services | Peer-reviewed |
| 88 | Pachanee, C. (2009). Implications on Public Health from Mode 2 Trade in Health Services: Empirical Evidence (Research report). Kobe: World Health Organization. | Trade in services | Research report |
| 89 | Pachanee, C., & Wibulpolprasert, S. (2003). Trade in health services and GATS: A case of Thailand (Research report). Ottawa: World Health Organization. | Trade in services | Research report |
| 90 | Pachanee, C., & Wibulpolprasert, S. (2004). Policy coherence between health related trade and health system development in Thailand (Research report). New Delhi: Trade and Health, WHO/SEARO. | Cross-cutting issue | Research report |
| 91 | Pachanee, C., & Wibulpolprasert, S. (2005). Making foreign direct investment in health services works to improve health care for the poor: The experiences from Thailand (Research report). Bangkok: International Health Policy Program. | Trade in services | Research report |

| | | | |
|---|---|---|---|
| 92 | Pachanee, C., & Wibulpolprasert, S. (2006). Incoherent policies on universal coverage of health insurance and promotion of international trade in health services in Thailand. Health Policy Plan, 21(4), 310-318. https://doi.org/10.1093/heapol/czl017 | Trade in services | Peer-reviewed |
| 93 | Pachanee, C., & Wibulpolprasert, S. (2008). MRAs for health professionals in ASEAN - initial movements for future freer flow of health professionals in ASEAN? (Research report). Bangkok: International Health Policy Program. | Trade in services | Research report |
| 94 | Pannarunothai, S., & Suknark, K. (2005). The impact of the free trade agreement on trade in health services on Thailand (Research reprot). Bangkok: Thammasat University. | Trade in services | Research report |
| 95 | Pattharapinyophong, W., & Sukpatch, K. (2017). The development of medical tourism in Thailand. Liberal Arts Review, 24. | Trade in services | Peer reviewed |
| 96 | Pavananunt, P. (2011). Illicit cigarette trade in Thailand. Southeast Asian J Trop Med Public Health, 42(6), 1531-1539. | Trade in goods | Peer-reviewed |
| 97 | Phueksuwan, T. (2014). Compulsory licensing policy implementation in Thailand (Doctoral dissertation). National Institute of Development Administration. | Intellectual property | Dissertation |
| 98 | Pipattanachat, V. (2017). Issues of civil liability for compensation from the tobacco industry according to FCTC Section 19 of Thailand. Public Health & Health Laws Journal, 3(3), 388-400. | Dispute settlement | Peer reviewed |
| 99 | Pocock, N., & Phua, K. (2011). Medical tourism and policy implications for health systems: a conceptual framework from a comparative study of Thailand, Singapore and Malaysia. Globalization And Health, 7(1), 1-12. https://doi.org/Globalization and Health 7(1):12 | Trade in services | Peer reviewed |
| 100 | Potisathian, P. (2009). Flexible mesures under article 21 of TRIPS agreement and access to medicine: The study of Thailand's compulsory licensing (Master's thesis). Chulalongkorn University. | Intellectual property | Thesis |
| 101 | Praditaukrit, S. (1993). The impact of patents Act B.E. 2535 on consumers, producers, and trade between Thailand and the United States: A case study of Pharmaceutical patents (Master's thesis). Kasetsart University. | Intellectual property | Thesis |
| 102 | Prakongsai, P., Akaleephan, C., Pagaiya, N., & Pagaiya, C. (2012). Policy recommendations on becoming an international medical hub (Research report). Bangkok: International Health Policy Program. | Trade in services | Research report |
| 103 | Prommarat, J. (2011). Legal problems on the interpretation of patent protection exclusion with respect to methods of diagnosis treatment or cure of human diseases in Thailand (Master's thesis). Dhurakij Pundit University. | Intellectual property | Thesis |
| 104 | Rerkrujipimol, J., & Assenov, I. (2011). Marketing strategies for promoting medical tourism in Thailand. Journal Of Tourism, Hospitality & Culinary Arts, 3(2), 95-105. | Trade in services | Peer reviewed |
| 105 | Rermkul, C. (2008). The economic impact of the compulsory licensing of anti HIV drugs in the case of Thailand (Master's thesis). Khon Kaen University. | Intellectual property | Thesis |
| 106 | Ritwatthanavanich, N. (2017). The scenario model of the aesthetic plastic surgery business for preparing a Thai population culture wave on medical tourism (Doctoral dissertation). Rajamangala University of Technology Phra Nakhon. | Trade in services | Dissertation |
| 107 | Rosenberg, S. (2014). Asserting the primary of health over patent rights: A comparative study of the processes that led to the use of compulsory licensing in Thailand and Brazil. Developing World Bioethics, 14(2), 83-91. https://doi.org/10.1111/dewb.12050 | Intellectual property | Peer-reviewed |

| | | | |
|---|---|---|---|
| 108 | Rujikittioangsuthon, S. (2010). The legal issues of pharmaceutical patent: Case study on licensing agreement (Master's thesis). Mae Fah Luang University. | Intellectual property | Thesis |
| 109 | Saktontai, S. (2007). The Key determinant factors of pharmaceutical industry in Thailand under the FTA Thailand-U.S.A. (Master's thesis). University of the Thai Chamber of Commerce. | Trade in goods | Thesis |
| 110 | Sasanapitak, A., Kongpradit, S., & Thomrongajariyakul, J. (2016). The measurement synthesis of limiting alcohol drinking access in the group of ASEAN countries (Research report). Bangkok: Center of Alcohol Studies. | Trade in goods | Research report |
| 111 | Senaveenin, P. (2005). Problems and prospects concerning the protection of drug patents under the legal frameworks of the World Trade Organization: Access to antiretroviral medicine (ARV) as a case study (Master's thesis). Prince of Songkla University. | Intellectual property | Thesis |
| 112 | Siahpush, M., Borland, R., Fong, G., Elton-Marshall, T., Yong, H., & Holumyong, C. (2011). Socioeconomic differences in the effectiveness of the removal of the "light" descriptor on cigarette packs: Findings from the international tobacco control (ITC) thailand survey. Int J Environ Res Public Health, 8(6), 2170-2180. https://doi.org/10.3390/ijerph8062170 | Trade in goods | Peer reviewed |
| 113 | Siasiriwattana, S., Kanajaroen, A., Rathnaarmol, P., Singkaow, S., Kangwanlurk, R., Audnakitthi, S., & Prasitthimat, J. (2020). The impact of medical services on free trade agreement between Thailand and the United States of America (Research report). Bangkok: The Secretariat of the Senate. | Trade in services | Research report |
| 114 | Siriwattana, S. (1996). The influence of transnational corporations in determining intellectual property policy in Thailand: The casharmaceutical productse of p (Master's thesis). Chulalongkorn University. | Intellectual property | Thesis |
| 115 | Sitasuta, P. (2015). World trade organization agreements and the scheme for tobacco plain packaging (Master's thesis). Chulalongkorn University. | Intellectual property | Thesis |
| 116 | Skees, S. (2007). Thai-ing up the TRIPS agreement: Are compulsory licenses the answer to Thailand’s AIDS epidemic?. Pace International Law Review, 19(2), 233-285. | Intellectual property | Peer reviewed |
| 117 | Smith, R., Chanda, R., & Tangcharoensathien, V. (2009). Trade in health-related services. Lancet, 14(373), 593-601. https://doi.org/10.1016/S0140-6736(08)61778-X | Trade in services | Peer reviewed |
| 118 | Sonpiam, S. (2015). Medical toursim in Thailand: A cross-cultural study of medical tourists' decision-making factors (Master's thesis). Prince of Songkla University. | Trade in services | Thesis |
| 119 | Sornprachoom, P. (1991). Guidekines for pharmacuetical product patent protection in Thailand (Master's thesis). Chulalongkorn University. | Intellectual property | Thesis |
| 120 | Sunanta, S. (2020). Globalising the Thai 'high-touch' industry: exports of care and body work and gendered mobilities to and from Thailand. Journal Of Ethnic And Migration Studies, 46(8), 1543-1561. https://doi.org/10.1080/1369183X.2020.1711568 | Trade in services | Peer-reviewed |
| 121 | Sungayuth, C., & Pachanee, C. (2014). Impacts of ASEAN Mutual Recognition Arrangements (MRAs) for health professionals on the Health System in Thailand: The analysis of the experience from the European Union. Health Systems Research Institute (HSRI), 8(1), 15-26. | Trade in services | Peer reviewed |
| 122 | Supakankunti, S., & Herberholz, C. (2015). Transforming the ASEAN economic community (AEC) into a global services hub: Enhancing the competitiveness of the health services sector in Thailand. Journal Of Business And Economics, 6(6), 1128-1135. https://doi.org/10.15341/jbe(2155-7950)/06.06.2015/009 | Trade in services | Peer reviewed |

| | | | |
|---|---|---|---|
| 123 | Supakankunti, S., Janjaroen, W., Tangphao, O., Ratanawijitrasin, S., Kraipornsak, P., & Pradithavanij, P. (2001). Impact of the World Trade Organization TRIPS Agreement on the pharmaceutical industry in Thailand. Bulletin Of The World Health Organization, 79(5), 461-470. https://doi.org/10.1590/S0042-96862001000500013 | Intellectual property | Peer reviewed |
| 124 | Suwanwachim, G., Suwannawach, P., Buranakul, A., & Chetupong, P. (2016). Creating medical tourism business competitive advantage in Thailand (Research report). Bangkok: Rajamangala University of Technology Phra Nakhon. | Trade in services | Research report |
| 125 | Suwanwela, C., Junjaroen, W., Sitthiamorn, J., Supkarnjanakanthi, S., Ratthanawejitthasil, S., & Pinitpuwadon, K. et al. (1999). A preliminary study on social impact and public health system in Thailand, and preparation from the free trade agreement on trade in health service. (Research report). Bangkok: College of Public Health Sciences, Chulalongkorn University. | Trade in services | Research report |
| 126 | Taechawatchananont, N. (2013). Potential analysis of medical tourism business in Chiang Mai province (Independent study). Chiang Mai Unviersity. | Trade in services | Independent study |
| 127 | Tammapitakkul, P., & Tammapitakkul, S. (2014). Patent law and problem of access to pharmaceutical product: A case study Thailand. EAU Heritage Journal Social Science And Humanity, 4(3), 109-123. | Intellectual property | Peer-reviewed |
| 128 | Tanchinwuttanakul, K. (2016). Protection of public health under the Model Bilateral Investment Agreement (BIT) of Thailand: the Case of tobacco (Research report). Prague: Faculty of Law, Charles University. | Dispute settlement | Research report |
| 129 | Tangwinit, N. (2008). Free trade and fair trade : A case study of the pharmaceutical patent in Thailand (Master's thesis). Chulalongkorn University. | Intellectual property | Thesis |
| 130 | Tantivess, S., Kessomboon, N., & Laongbua, C. (2007). The role of key stakeholders in the introduction of the government use of patents for essential medicines in Thailand. Journal Of Health Systems Research, 2(3), 350-359. | Intellectual property | Peer reviewed |
| 131 | Taychakhoonavudh, S. (2009). Effect of the compulsory license on the public provision of the announced medicine in Thailand (Master's thesis). Chulalongkorn University. | Intellectual property | Thesis |
| 132 | Thaiprayoon, S., & Smith, R. (2014). Capacity building for global health diplomacy: Thailand's experience of trade and health. Health Policy And Planning, 30, 1118-1128. https://doi.org/10.1093/heapol/czu117 | Cross-cutting issue | Peer-reviewed |
| 133 | Thanitcul, S. (2008). TRIPS Agreement on the compulsory licensing on medicine in Thailand. The Journal Of Faculty Of Law Chualalongkorn University, 27(1). | Intellectual property | Peer reviewed |
| 134 | Thanitcul, S., & Braslow, M. (2013). Compulsory licensing of chronic disease pharmaceuticals in Thailand. Thai Journal of Pharmaceutical Sciences, 37(2), 61-83. | Intellectual property | Peer-reviewed |
| 135 | Thipkongkar, P. (2008). The impact of intellectual property negotiations regarding the US-Thailand FTA draft for the pharmaceutical industry (Master's thesis). Ramkhamhaeng University. | Intellectual property | Thesis |
| 136 | Trakoonmechokchai, P. (2005). Impacts of free trade agreement on research and development of pharmaceutical products in Thailand (Master's thesis). Chulalongkorn University. | Trade in goods | Thesis |
| 137 | Trethasayuth, S. (2004). The effect of intellectual property protection on international trade : A case study of Thailand (Master's thesis). Thammasat University. | Intellectual property | Thesis |
| 138 | Tungbuntina, P. (2005). The impact of medical patent on medical import and export of Thailand (Master's thesis). Kasetsart University. | Intellectual property | Thesis |

| | | | |
|---|---|---|---|
| 139 | Watanapa, P. (2007). To make Thailand become a medical hub and center of medical tourism of Asia: Opportunity, critical success factors and effects on Thailand economy. Field economics (Research report). Bangkok: National Defence College of Thailand. | Trade in services | Research report |
| 140 | Wathanapisit, U. (2009). Legal protection of pharmaceutical registration data: data exclusivity (Master's thesis). Chulalongkorn University. | Intellectual property | Thesis |
| 141 | Wibulpolprasert, S., & Pachanee, C. (2008). Addressing the internal brain drain of medical doctors in Thailand: the story and lesson learned. Global Social Policy, 8(1), 12-15. https://doi.org/10.1177/14680181080080010104 | Trade in services | Peer reviewed |
| 142 | Wibulpolprasert, S., Pachanee, C., Pitayarangsarit, S., & Hempisut, P. (2004). International service trade and its implications for human resources for health: A case study of Thailand. Human Resources For Health, 2(10), 1-12. https://doi.org/10.1186/1478-4491-2-10 | Trade in services | Peer reviewed |
| 143 | Wichasin, P., Sangsue, P., & Gladgaew, N. (2017). Strategies for enhancing expenditure and creating value in the medical tourism industry in order to extend the stay and encourage repeat visiting of international medical tourists in Thailand (Research report). Bangkok: Saun Dusit University. | Trade in services | Research report |
| 144 | Wilson, A. (2011). Foreign bodies and national scales: Medical tourism in Thailand. Body & Society, 17(2&3), 121-137. https://doi.org/10.1177/1357034X11400923 | Trade in services | Peer-reviewed |
| 145 | Wong, K., & Musa, G. (2012). Medical tourism in Asia: Thailand, Singapore, Malaysia, and India. | Trade in services | Peer reviewed |
| 146 | Wongchum, R., LeSeure, P., Songsiri, O., Janthanakul, P., Charoenkitkarn, V., & Akaleephan, C. (2018). Foreigners employed as hospital workforce in Thailand. Journal Of Health Systems Research, 12(2), 292-305. Retrieved 5 December 2020, from. | Trade in services | Peer reviewed |
| 147 | Wongkit, M., & McKercher, B. (2013). Toward a typology of medical tourists: A case study of Thailand. Tourism Management, 38, 4-12. https://doi.org/10.1016/j.tourman.2013.02.003 | Trade in services | Peer-reviewed |
| 148 | Wongkit, M., & McKercher, B. (2016). Desired attributes of medical treatment and medical service providers: A case study of medical tourism in Thailand. Journal Of Travel & Tourism Marketing, 33(1), https://doi.org/14-27. https://doi.org/10.1080/10548408.2015.1024911 | Trade in services | Peer-reviewed |
| 149 | Yamabhai, I., & Smith, R. (2012). A review of the health and economic implications of patent protection, with a specific focus on Thailand. Health Research Policy And Systems, 10(24), 1-18. https://doi.org/10.1186/1478-4505-10-24 | Intellectual property | Peer reviewed |
| 150 | Yamabhai, I., Mohara, A., Tantivess, S., Chaisiri, K., & Teerawattananon, Y. (2011). Government use licenses in Thailand: An assessment of the health and economic impacts. Globalization And Health Volume, 7(28). https://doi.org/10.1186/1744-8603-7-28 | Intellectual property | Peer-reviewed |
| 151 | Yamabhai, I., Santatiwongchai, B., & Akaleephan, C. (2017). Assessing the impact of the Thailand-European union free trade agreement on trade and investment in Thailand. Health Economics & Outcome Research: Open Access, 31(1). https://doi.org/10.4172/2471-268X.1000.S1-106 | Intellectual property | Peer reviewed |
| 152 | Yamabhai, I., Sosom, J., & Pilasant, S. (2018). Expropriation of Projects that affecting the Environment and Health: A Case Study of Gold Mining (Research report). Bangkok: International Trade and Health Programme. | Dispute settlement | Research report |
| 153 | Ye, H. & Assenov, I. (2017). Mainland Chinese tourists' perception of medical tourism in Thailand. Research Journal Of Social Sciences, 10(2), 8-13. | Trade in services | Peer reviewed |

| | | | |
|---|---|---|---|
| 154 | Yoongthong, T. (2012). Social cost of market exclusivity extension for patented medicines in Thailand : Analysis of the effect of trips-plus provisions (Doctoral dissertation). National Institute of Development Administration. | Intellectual property | Dissertation |
| 155 | Yoongthong, T. (2015). Welfare effect of market exclusivity extension for patented medicines in Thailand: Analysis of the effect of TRIPS-Plus provisions. Journal Of Population And Social Studies, 23(2), 193-213. | Intellectual property | Peer-reviewed |
| 156 | Zhao, X. (2017). Factors influencing Chinese medical tourists' satisfaction with medical services in Thailand (Master's thesis). Chulalongkorn University. | Trade in services | Thesis |
